# Gene Expression Alterations during Development of Castration-Resistant Prostate Cancer Are Detected in Circulating Tumor Cells

**DOI:** 10.3390/cancers12010039

**Published:** 2019-12-21

**Authors:** Andreas Josefsson, Karin Larsson, Eva Freyhult, Jan-Erik Damber, Karin Welén

**Affiliations:** 1 Department of Urology, Sahlgrenska Cancer Center, Institute of Clinical Sciences, Sahlgrenska Academy, University of Gothenburg, 405 30 Gothenburg, Sweden; 2 Department of Surgical and Perioperative Sciences, Urology & Andrology, Umeå University, 901 87 Umeå, Sweden; 3 Wallenberg Center for Molecular Medicine, Umeå University, 901 87 Umeå, Sweden; 4 Department of Medical Sciences, National Bioinformatics Infrastructure Sweden, Science for Life Laboratory, Uppsala University, 751 85 Uppsala, Sweden

**Keywords:** CTC, hormone-sensitive prostate cancer, CRPC, liquid biopsy, biomarker, resistance mechanisms

## Abstract

Development of castration-resistant prostate cancer (CRPC) is associated with alterations in gene expression involved in steroidogenesis and androgen signaling. This study investigates whether gene expression changes related to CRPC development can be identified in circulating tumor cells (CTCs). Gene expression in paired CTC samples from 29 patients, before androgen deprivation therapy (ADT) and at CRPC relapse, was compared using a panel including 47 genes related to prostate cancer progression on a qPCR platform. Fourteen genes displayed significantly changed gene expression in CTCs at CRPC relapse compared to before start of ADT. The genes with increased expression at CRPC relapse were related to steroidogenesis, AR-signaling, and anti-apoptosis. In contrast, expression of prostate markers was downregulated at CRPC. We also show that midkine (MDK) expression in CTCs from metastatic hormone-sensitive prostate cancer (mHSPC) was associated to short cancer-specific survival (CSS). In conclusion, this study shows that gene expression patterns in CTCs reflect the development of CRPC, and that MDK expression levels in CTCs are prognostic for cancer-specific survival in mHSPC. This study emphasizes the role of CTCs in exploring mechanisms of therapy resistance, as well as a promising biomarker for prognostic and treatment-predictive purposes in advanced mHSPC.

## 1. Introduction

Metastatic castration-resistant prostate cancer (mCRPC) is responsible for most deaths from prostate cancer. CRPC develops during androgen deprivation therapy (ADT), the standard therapy for metastatic prostate cancer (PC). ADT inhibits the testicular production of testosterone resulting in low circulating levels and inhibited growth of androgen-dependent PC cells. However, androgen receptor signaling is crucial for most PCs, and several mechanisms for its sustained activity exist, enabling the relapse and growth of CRPC. Although CRPC is still lethal, today many life-prolonging therapies are in clinical use, and patients now live longer with CRPC than in the hormone-dependent phase of metastatic PC [1].

It is well established that increased expression of steroidogenic enzymes and constitutively active forms of the androgen receptor are characteristics of CRPC [2,3,4]. The steroidogenic enzymes confer an intratumoral steroid synthesis resulting in higher testosterone levels within tumors compared to the castrate levels present systemically during ADT [5]. In addition to increased steroid levels, the androgen receptor (AR) presents itself in several differently mutated or spliced variants with increased sensitivity, stability, or activity to maintain sufficient signaling for the PC cells to survive and proliferate [6,7,8,9].

Both these strategies can partly be targeted by the new generation of hormone-related drugs. Abiraterone acetate inhibits CYP17A1, which converts pregnenolone and progesterone to DHEA and androstenedione, respectively, both important precursors to testosterone [10]. Enzalutamide is a new-generation AR inhibitor that is more efficient compared to, for example, bicalutamide [11]. However, CRPC patients with expression of the constitutively active AR splice variant AR-V7 often display resistance to both these therapies [12,13]. In addition to AR-targeting drugs, other therapies such as chemotherapy (docetaxel, cabazitaxel), targeted radiation (radium-223), and to some extent immunotherapy (Sipuleucel-T) contribute to the prolonged survival of patients with CRPC [14].

To achieve the most efficient use of different drugs for the benefit for the patients, individualized treatment protocols are needed. In metastatic disease, accurate characterization of tumor biology is challenging. However, circulating tumor cells in the blood stream have been shown to mirror metastatic phenotype [15], and analysis of circulating tumor cells (CTCs) may be a tool to get information for clinical decisions.

In the present study, we isolated CTCs from patients with advanced metastatic PC undergoing ADT to investigate whether the changes occurring in the tumors can be identified in CTCs. The correlation of CTC gene expression before therapy with progression on ADT was also investigated.

## 2. Results

Forty patients with metastatic hormone-sensitive prostate cancer (mHSPC) were included, and 37 of these displayed signals for at least two of the genes analyzed, not related to the leucocyte contamination, and were defined as CTC positive and included in the study. The median follow-up time for all 40 included patients was 27.5 months (range 0.7–72.8 months). The three CTC negative patients did not have detected metastases at start of ADT, and their follow-up times were 23.5, 43.2, and 62.1 months. For the 37 CTC positive patients included in the survival analysis, the follow-up times, cancer-specific survival (CSS), and time to CRPC can be found in Table 1.

For the 28 of these patients that died of PC within the study, the median time from ADT to PC-death was 17.3 months (Q1: 10.9, Q3: 32.1), and the time from CRPC to PC-death was 10.5 months (Q1: 5.1, Q3: 27.5). For the patients still alive at last follow-up (*n* = 7, two died of other causes), the times from ADT and CRPC to last follow-up were 45.1 months (Q1: 38.7, Q3: 52.9) and 40.6 months (Q1: 33.2, Q3: 53.3), respectively. At CRPC relapse, eight of the 37 patients were either CTC negative (*n* = 3) or were not sampled (*n* = 5), leaving 29 patients for comparisons of CTC gene expression alterations during ADT.

Of the 47 assays included in the PC-panel used for detection of gene expression, seven genes (*ESR1*, *ESR2*, *PTCH1*, *CYP11A1*, *CYP17A1*, *CYP19A1*, and *MET1*) were excluded from analysis, since they could only rarely be detected in the CTCs sampled. Six of the genes (*MYC*, *TP53*, *ANXAR2*, *AKT*, *ALDHA1*, and *RUNX2*) were excluded since they were strongly correlated to *CD45* (statistically significant correlation (*p* < 0.05) with a correlation coefficient >0.5), thus the signal may come from the contaminating population of white blood cells, making interpretations about expression in CTCs difficult. In addition, the four control genes (*GAPDH*, *GUSB*, *CD44*, and *CD45*) were not included in the analysis. The numbers of detected signals for the remaining 30 genes are presented in Table 2.

### 2.1. Alterations in Gene Expression during ADT

Changes in gene expression in CTCs during ADT were investigated by a pair-wise comparison of the ΔCq values of the CTC samples before start of ADT and at CRPC relapse. Missing signals due to undetected gene expression were replaced with a calculated ΔCq value representing low expression when appropriate (see Methods section). A pair consisting of two calculated ΔCq replaced for missing signals was excluded from analysis, resulting in a varying number of patients included in the analysis for different genes (Table 2).

**Figure 1:**
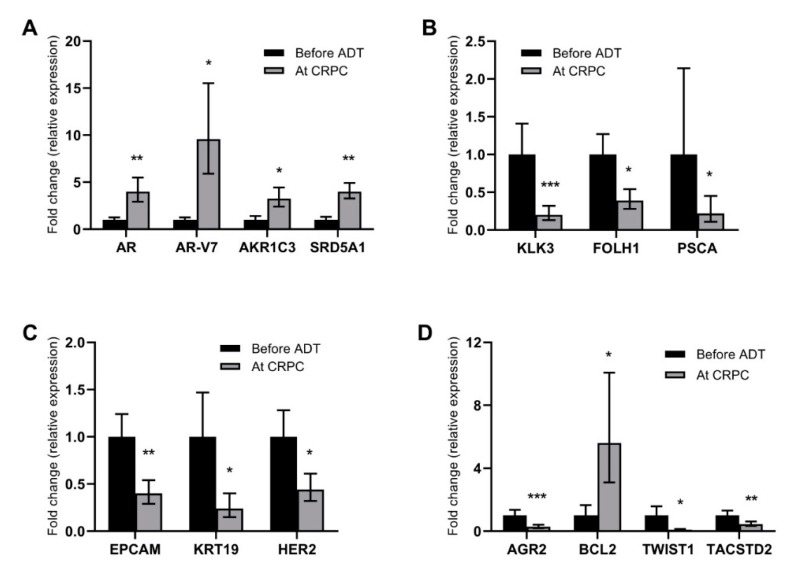
Genes with altered gene expression at CRPC relapse. Graphs illustrate differences in gene expression levels in paired CTCs sampled before ADT (black bars) and at CRPC relapse (grey bars). Differences are displayed as relative changes (fold change) in relation to levels before ADT for (**A**) genes related to androgen signaling; (**B**) prostate markers; (**C**) genes related to epithelial phenotype; and (**D**) other genes with altered expression levels. Bars represent mean fold change ± standard error of the mean (SEM); the black bars for before ADT always shows 1 ± SEM by definition of the method. Statistically significant differences are denoted with * = *p* < 0.05, ** = *p* < 0.01, and *** = *p* < 0.001.

In the comparative analysis, 14 of the 30 genes displayed altered expression levels at CRPC relapse compared to before start of ADT. Increased expression was detected for *AR* (*p* < 0.01), *AR-V7* (*p* < 0.05) and the steroidogenic enzymes *AKR1C3* (*p* < 0.05) and *SRD5A1* (*p* < 0.01) (Figure 1A). In contrast, the expression of the prostate cancer marker genes *KLK3* (*p* < 0.01), *FOLH1* (*p* < 0.05), and *PSCA* (*p* < 0.05) was decreased in CTCs at CRPC relapse (Figure 1B). Expression of genes related to an epithelial phenotype (*EPCAM* (*p* < 0.01), *KRT19* (*p* < 0.05), and *HER2* (*p* < 0.05)) was found to be decreased at CRPC relapse (Figure 1C). Other genes with altered expression levels were the anti-apoptotic *BCL2* (upregulated; *p* < 0.05), the epithelial-to-mesenchymal transition marker *TWIST1* (downregulated; *p* < 0.01), the stem cell marker *TACSTD2* (downregulated; *p* < 0.05), and *AGR2*, an androgen-regulated gene associated with metastasis in PC [16,17,18] (downregulated; *p* < 0.01) (Figure 1D).

### 2.2. Gene Expression in CTCs as Prognostic Markers for Survival

The possible prognostic information of gene expression levels in CTCs was assessed by Cox regression analysis relating the gene expression in CTCs before start of ADT to either time to development of CRPC or cancer-specific survival (CSS). To ensure that no false correlations due to an exaggerated estimated low value would be detected, a stricter cut-off level for substituted low values was applied for this analysis. Based on the highest mCq at which a specific gene could be detected, the cut-off was set one Cq lower, i.e., missing signals were only replaced and included in samples with a higher CTC content (one mCq lower) than the one representing the detection limit for the specific gene. Thus, the number of data points included varies among the genes analyzed (Table 2), and a lack of correlation could be a consequence of few data points in the analysis for certain genes.

The only gene that was significantly associated to time to development of CRPC was *EPCAM* (*p* = 0.012). This is most likely due to the fact that EPCAM is the epitope used for capture of the CTCs, and that it therefore, also after normalization, largely reflects the CTC content, which is expected to correlate to prognosis. mCq, the surrogate value for CTC content in this study, was strongly associated to a short time to development of CRPC (*p* = 0.0005) (Table 3). Other genes were not associated to time to CRPC.

Similarly, both *EPCAM* (*p* = 0.002) and mCq (*p* = 0.0003) were significantly associated to CSS with Cox regression statistics. In addition, a high *MDK* (Midkine) expression in CTCs was associated to a poor CSS (*p* = 0.008). The EMT-associated gene *TWIST1* was inversely associated to CSS (*p* = 0.05). No other genes were associated to CSS. None of the genes identified as significant in the univariable analysis remained prognostic when analyzed together in multivariable analysis.

**Figure 2:**
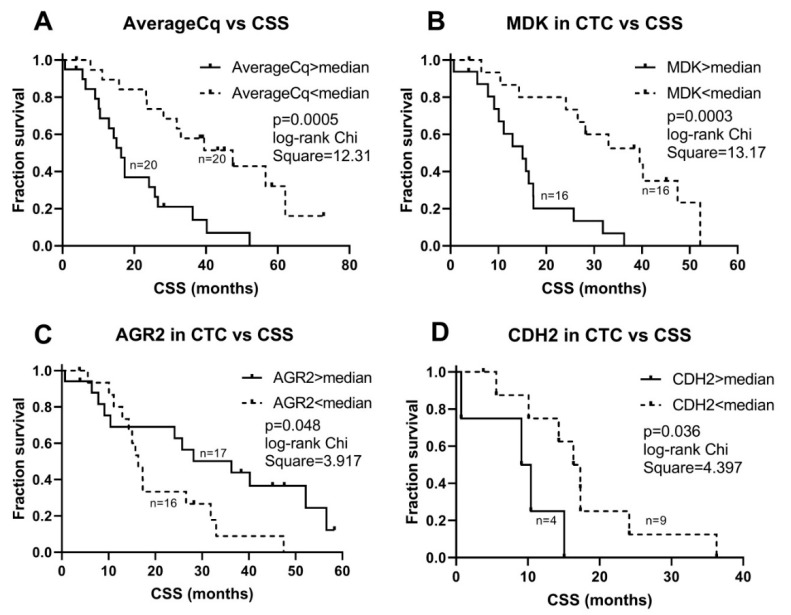
Survival plots of dichotomized CTC acquired parameters. Graphs illustrate Kaplan–Meier statistics of (**A**) average signal strength (mCq) as a proxy for CTC content, (**B**) midkine (MDK) gene expression in CTCs (normalized to AverageCq), (**C**) AGR2 expression in CTCs (normalized to Average Cq), and (**D**) N-cadherin (CDH2) expression in CTCs (normalized to Average Cq), in relation to cancer-specific survival (CSS). Parameters are grouped as higher or lower than their respective median values.

No parameters were significantly associated with time to CRPC using Kaplan–Meier statistics. Using non-parametric Kaplan–Meier statistics to verify the associations with CSS, *EPCAM* (*p* = 0.046), mCq (*p* = 0.0005), and *MDK* (*p* = 0.0003) were confirmed to have statistically significant associations (Figure 2), while *TWIST1* did not (*p* = 0.252). In addition, the EMT-related gene *CDH2* and the androgen-regulated gene *AGR2* were found to be significantly associated with CSS (*p* = 0.036, and *p* = 0.048, respectively) (Figure 2). The expression of the highly prostate-cancer-related genes *AR*, *AR-V7* and *FOLH1* was not associated to survival.

## 3. Discussion

In CRPC, CTCs have been shown to be a good biomarker for prognosis, based on their enumeration, and treatment-predictive purposes, based on their expression of AR-V7 [12,19]. Their expression pattern also reflects the phenotype of skeletal metastases [15]. However, the potential of CTCs as a source for phenotypic information also in the hormone-sensitive stage of the disease has not been extensively investigated. The present study provides data emphasizing the value of CTC characterization in hormone-sensitive PC both for phenotypic investigation of disease progression and for biomarker purposes.

This is the first study that investigates consecutive CTC samples during the progression from hormone-sensitive to castration-resistant prostate cancer. In the pairwise analysis of these samples, we identified a number of genes, the expression of which changed during the development of CRPC. In line with previous studies on tumor tissue, the CTC expression of genes in the androgen signaling pathway was increased in CRPC compared to before start of ADT. Expression of both AR and its constitutively active splice variant AR-V7 are increased in CRPC tissue [3,4,20] reflecting an increased androgen sensitivity and AR signaling in the CRPC state. Increased expression of the steroidogenic enzyme AKR1C3 has been reported in CRPC tissue [4,21], pointing out conversion of adrenal steroid precursors as a resistance mechanism to ADT and promoting intratumoral androgen synthesis enabling sustained AR signaling despite castrate levels of testosterone in the circulation. SRD5A1 converts testosterone to dihydrotestosterone (DHT) enabling more potent activation of the AR. Although previous studies have showed the increased importance of SRD5A1 compared to SRD5A2 in advanced PC [22] as well as increased expression of SRD5A1 in metastatic PC compared to primary tumors [3,21,23,24], this study is the first to suggest further increased expression of SRD5A1 specifically in CRPC. While most other studies compared unpaired tumor tissue samples of different clinical stages, the present study used paired samples taken consecutively from the patients, which may explain this discrepancy. However, the biological importance of this finding is somewhat hard to define, since it has been demonstrated that despite increased intratumoral levels of testosterone in CRPC, correspondingly high DHT levels have not been detected [3,5]. Although PSA (*KLK3*) is the established biomarker for monitoring of clinical response to ADT, its expression in individual cells is known to be lower in dedifferentiated PC cells [25,26], which is in line with the decreased *KLK3* expression in CTCs from CRPC in the present study. Genes encoding other prostate antigens, *FOLH1* (PSMA) and *PSCA*, were also less expressed in CTCs at CRPC relapse, possibly representing the dedifferentiation process during disease progression. Taken together, the biological relevance of these observations of gene expression changes in CTCs strongly suggests that CTC sampling and analysis represent a useful tool for further exploring the molecular changes underlying the development of CRPC, as well as disease monitoring in the clinical setting.

Differences in gene expression in CTCs from hormone-naïve patients and patients with CRPC have not been extensively studied previously. In an unpaired analysis of single CTCs from eight patients, an increased expression of genes related to epithelial-to-mesenchymal transition (EMT) was observed [27]. A similar pattern was also seen in a study comparing CTCs from high-risk prostate cancer patients before and after surgery or radiotherapy [28]. In the present study using paired samples, there was no indication of an increase in EMT at the CRPC stage, despite substantial evidence of such in prostate cancer tissues [29,30]. This discrepancy may originate in different ways to isolate CTCs or to normalize the expression signals in the different studies on CTCs. The absence in CTCs of the observed EMT expression in tissue may also be related to a general difference between cells in tissue and cells released into the circulation.

There are two main issues that need to be addressed when evaluating differences in gene expression levels between CTC samples. First, there is the problem of finding a good way to normalize the gene expression to the number of CTCs, to be able to distinguish between different gene expression levels per se and a different detected signal due to the number of CTCs present in the sample. This is especially important for survival studies, since it is well known that a high CTC load corresponds to a poor prognosis, and thus a strong non-normalized gene expression signal could simply reflect the CTC load, and any conclusions on the importance of the specific gene may be false. In the present study, we used an approach to normalize using the average signal strength of all studied genes in a sample (excluding the genes significantly correlated to the leukocyte marker CD45, most likely representing the leukocyte contamination in the sample). The second problem is how to interpret missing signals, i.e., when expression of a gene could not be detected, a common problem when analyzing CTCs. This can of course be the result of a true low expression of the gene in question; however, it could also be due to a too low CTC content for the specific assay to detect and amplify any cDNA. Thus, for avoiding false low expression values, a cut-off for when to allow substitution of missing signals with low expression values has to be defined based on the CTC content of the sample and the sensitivity for the individual gene expression assays. In this study, for each individual gene, samples with lower CTC content than the one with the calculated lowest CTC content in which a signal could be detected were excluded from the analysis. In the survival analyses, an even stricter cut-off was applied to minimize any contribution from patients with a good prognosis mostly due to a low CTC content.

Prognostic studies using gene expression in CTCs have mainly been performed in CRPC, where especially the detection of AR-V7 has shown clinical potential in identifying patients with lower chance of benefitting from targeting of the androgen signaling axis by abiraterone acetate or enzalutamide [12,13]. It has also been demonstrated that AR-V7 mRNA directly from whole blood can be used for that purpose. However, all AR splice variants are not tumor-specific [31], making CTCs a more accurate source for disease prognostication, especially in low-volume disease. The multiplex gene expression analysis used in the present study aimed to demonstrate the potential of assessment of a broad range of prostate-cancer-related gene expression in CTCs for their potential value in disease prognostication. CTC content, estimated as average signal strength, showed a strong association with cancer-specific survival, as expected based on previous studies on CTC enumeration as a prognostic marker in prostate cancer [19,32]. Among the genes in our expression panel, MDK emerged as the most prognostic for cancer-specific survival. High MDK expression in CTCs before ADT was significantly associated to short CSS even after normalization for CTC content. MDK is a chemokine that is upregulated in CRPC and associated to neuroendocrine differentiation [33] and as secreted protein has a strong prognostic potential measured in plasma from patients with hormone-sensitive metastatic prostate cancer (Nordin et al., unpublished). In contrast to MDK, expression of AGR2 is induced by androgens [34] and was found to be inversely correlated to cancer-specific survival. EPCAM was also associated to survival, probably indicating that normalization did not fully eliminate the fact that CTCs were isolated based on their EPCAM expression in the present study. High expression of the EMT-related cell adhesion molecule N-cadherin (CDH2) was associated to a poor prognosis. N-cadherin is tightly connected to an invasive phenotype and has been associated to metastasis in prostate cancer [35,36]. Interestingly, high expression of another gene involved in EMT, the transcription factor TWIST1, was instead suggesting a good prognosis. This may seem contradictory, but the co-expression with N-cadherin may not be mechanistically linked in AR-positive cells. Our previous studies indicate a TWIST1-independent upregulation of N-cadherin in metastatic CRPC in vitro [36], as well as increased expression of *TWIST1* in LNCaP cells with low migration and poor colony formation capacity due to silencing of RGS2 [37]. In addition, recent studies by others show that AR-V7 and other splice variants may have direct transcriptional activity on *CDH2* without effects on *TWIST1* [38,39].

We have previously reported the prognostic value of detection of EGFR and ARV7 in hormone-sensitive metastatic prostate cancer, in a patient cohort partly overlapping the present study [40,41]. Our previous data could not be confirmed in this study where the multiplex gene expression analysis enables more careful normalization and accurate exclusion of samples with low CTC content.

In conclusion, this study demonstrates the property of CTCs to be a source of phenotypic information in relation to therapy response of metastatic disease. In addition, our data, showing that MDK expression in CTCs is strongly associated to poor prognosis of metastatic PC, highlight both the importance of MDK itself and the potential of profiling of CTCs as a prognostic and treatment-predictive tool for personalized medicine.

## 4. Materials and Methods

### 4.1. Patients

Forty patients with high probability of primary metastatic disease, median age 75 years, from the Department of Urology, Sahlgrenska University Hospital, Gothenburg, Sweden, were included between 2012 and 2016. Eligible patients were castration-naïve men presenting with PSA higher than 80 ng/mL or metastatic PC intended for ADT. A bone scan was performed on all patients. The patients underwent either medical (*n* = 38) or surgical castration (*n* = 2). Patients with medical castration had either GnRH-analogue with flair prophylaxis (antiandrogen for 4 weeks; *n* = 31) or GnRH-antagonist for one month followed by GnRH-analogue (*n* = 7).

Thirty-nine patients relapsed with CRPC within the study follow-up, and one died an unrelated death before any relapse criteria were met. CRPC relapse was defined according to the European Association of Urology definition (*n* = 30), based on skeletal related events or need for palliative radiation (*n* = 7), or due to PC-related death despite ongoing ADT before other CRPC criteria were met (*n* = 2). CSS was defined as death after progression of disease. Blood for the CTC analyses was collected prior to the initiation of ADT and at CRPC relapse or closely after. The physicians participating in the study were blinded to the results of the CTC analysis. All subjects gave their informed consent for inclusion before they participated in the study. The study was conducted in accordance with the Declaration of Helsinki, and the protocol was approved by the Ethics Committee of Gothenburg (936-12).

### 4.2. CTC Isolation and cDNA Synthesis

CTCs were isolated from blood and detected using the AdnaTest ProstateCancerSelect/Detect (Qiagen Hannover GmbH, Langenhagen, Germany) as previously described [41]. Briefly, patient blood samples were collected immediately prior to surgery in AdnaCollect tubes and kept refrigerated (4 °C) for no more than 24 h until CTC isolation. CTCs were captured with EPCAM and HER2 antibody-conjugated magnetic beads and lysed before mRNA was isolated with oligo-dT-conjugated magnetic beads and transcribed into cDNA.

### 4.3. Gene Expression Profiling

Two µL of cDNA samples were preamplified using the TATAA PreAmp Primer Mix and TATAA PreAmp GrandMaster^®^ Mix (Cat. No. #TA05, TATAA Biocenter, Gothenburg, Sweden). Preamplification was performed in a thermocycler (T100, BioRad, Hercules, CA, USA). Preamplification with no template control and human gDNA sample (concentration 0.5 ng/μL, TATAA Biocenter, Gothenburg, Sweden) were also included. The preamplified samples were spun down (to pellet the magnetic beads), and a fraction of the supernatant was moved to a separate tube and diluted 10×. The diluted samples were analyzed with 47 assays specifically designed for this study (now available in the GrandPerformance CTC Assay Panel (TATAA Biocenter, Gothenburg, Sweden)) and ValidPrime™ assay (TATAA Biocenter, Gothenburg, Sweden). For assay details see Table S1. The qPCR was performed using TATAA Probe GrandMaster^®^ Mix Low ROX (TATAA Biocenter, Gothenburg, Sweden) and GE 96.96 Dynamic Array™ Sample & Assay Loading Reagent Kit (P/N 85000802-R, Fluidigm, South San Francisco, CA, USA). Preamplification no template control (preAmp NTC) and no template control (NTC) for the qPCR were included. The qPCR was performed on BioMark (Fluidigm, South San Francisco, CA, USA) using the 96.96 Dynamic Array™ IFC (Integrated Fluidic Circuit). All samples (including NTCs and gDNA) were analyzed in duplicates.

### 4.4. Definition of CTC Load as Basis for Normalization and Handling of Missing Signals

Cycle of quantification (Cq) values for the individual genes were correlated to Cq values for CD45 as a marker for leucocyte contamination. Genes with a *p*-value less than 0.05 and a Spearman correlation coefficient above 0.5 (*AKT2*, *ALDH1A1*, *ANXA2R*, *CD44*, *GAPDH*, *GUSB*, *MYC*, and *RUNX2*) were removed from further analyses on the assumption that they largely represent gene expression in the contaminating leucocyte population.

For all other genes, missing signals (i.e., not detected with PCR) were imputed with Cq_max,g_ + 1, where Cq_max,g_ is the highest detectable Cq value for gene g, and an average Cq (mCq_k_) was calculated for each individual sample k. Using these mCq values, a ΔCq was calculated for all detected signals (ΔCq = Cq−mCq). Originally missing signals were reimputed with ΔCq_max,g_ + 1, i.e., one cycle higher than the highest identified ΔCq for that specific gene, g. A missing Cq value means that there were no detectable levels of the gene in the sample. There can of course be several different reasons for this; either there are insufficient cells in the sample, or the expression of the gene is zero or very low. To ensure that missing signals were not falsely interpreted as a low gene expression in samples where the CTC content was too low to enable signal detection, a cut-off was set for each gene, g, based on the sample, h, with the highest mCq in which the gene could be detected. For gene g, the cut off was calculated as: cut off-mCq_g_ = mCq_h,g_−((Cq_h,g_ − Cq_max,g_)/2), where mCq_h,g_ = Cq_max,g_ is the mCq for sample h and Cq_h,g_ is the Cq for gene g in sample h. Using this cut off, a missing value was only imputed and included in the analysis if its sample mCq was lower or equal to the cut-off. The cut-off calculations were based on approximately 230 CTC samples from prostate cancer patients, most of them not included in the analysis in the present study. A step-wise example of the calculations can be found as a supplementary file (Table S3).

### 4.5. Statistical Analysis

Differences between gene expression in CTCs before ADT and at CRPC relapse were statistically evaluated using the Wilcoxon test, a paired rank-based non-parametric test. Survival analysis was performed using univariate Cox regression analysis and Kaplan–Meier analysis. A stricter cut-off was used for survival analysis than for the pair-wise comparisons. Samples with imputed values were only included in survival analysis if their mCq was one cycle lower (i.e., had a higher CTC content) than the mCq for the sample with the highest mCq in which the gene could be detected (mCq_h,g_). Cox regression analysis was performed on continuous data, and Kaplan–Meier log-rank analysis was performed on patients categorized based on expression levels above or below the median expression level of the specific gene. Genes for which more than 50% of values were imputed were excluded from Cox regression analysis (Table S2).

## 5. Conclusions

We show that liquid biopsies in the form of CTCs harbor treatment prognostic information regarding CSS in mHSPC. Furthermore, CTCs can provide deeper knowledge on treatment-induced expression changes, enabling identification of novel therapeutic targets.

## Figures and Tables

**Table 1:** Patient characteristics of 37 circulating tumor cell (CTC)-positive patients).

Clinical Variables	Number or Median (Q1; Q3)
**PSA before androgen deprivation therapy (ADT)**	590 ng/mL (270; 1900)
**Gleason sum**	
6–7	4
8	9
9–10	17
x	7
**Type of ADT**	
GnRH agonist	28
GnRH antagonist	7
Orchidectomy	2
**Time from ADT to CRPC relapse**	8.4 months (4.9; 12.9)
**Castration-resistant prostate cancer CRPC Therapy**	
Total androgen blockade (bicalutamide)	25
Abiraterone acetate or enzalutamide	11
Radium-223	9
Docetaxel or cabazitaxel	8
Cyclophosphamide	2
None	5
Time from ADT to prostate cancer death or last follow-up (cancer-specific survival, CSS) (*n* = 35 *)	25.7 months (13.6; 39.3)
**Total follow-up time (*n* = 37)**	24.1 months (11.1; 39.0)

* Two patients died of other causes.

**Table 2:** Gene expression signals detected and included in paired analysis.

Gene	Before ADT (*n* = 40) (A)		At CRPC Relapse (*n* = 32)		Pairs Included in Comparison (A vs. CRPC) **
	Detected Signals	Substituted * Missing Signals	Detected Signals	Substituted * Missing Signals	
*AGR2*	30	8	14	18	25
*AHR*	13	24	9	20	13
*AKR1C3*	23	17	21	11	24
*AR*	23	18	17	14	19
*ARV7*	9	26	10	8	12
*AURKA*	21	19	19	10	22
*BCL2*	16	23	17	11	21
*CDH1*	22	14	14	15	17
*CDH2*	4	30	4	24	4
*DDR1*	13	23	13	16	14
*EGFR*	12	9	8	9	9
*EMP2*	22	16	13	17	16
*EPCAM*	36	2	23	9	29
*FOLH1*	30	7	15	14	22
*UPA*	11	25	10	15	13
*HER2*	24	16	16	16	21
*KLK3*	33	5	21	11	27
*KRT19*	28	9	17	14	25
*MDK*	29	11	16	15	25
*POU5F1*	19	20	18	14	22
*PSCA*	17	20	9	21	14
*SNAI1*	10	19	4	22	9
*SPINK1*	20	20	12	20	18
*SRD5A1*	25	15	27	4	28
*TACSTD2*	29	9	18	8	24
*TOP2A*	26	13	18	8	21
*TP53*	34	6	28	4	28
*TUBB3*	21	15	14	16	21
*TWIST1*	21	17	11	21	19
*VEGFA*	24	16	17	15	23

* Missing signals in samples with too low CTC load for individual genes to be detected are not substituted. ** Pairs need to contain at least one detected signal to be included in analysis.

**Table 3:** Cox regression analysis for survival.

Parameter	Time to CRPC		Cancer-Specific Survival	
	*p*-Value	Hazard Ratio	*p*-Value	Hazard Ratio
		(95% Confidence Interval)		(95% Confidence Interval)
Average Cq	**0.0005**	1.22 (1.09–1.38)	**0.0003**	1.24 (1.10–1.39)
EPCAM	**0.012**	1.32 (1.06–1.65)	**0.002**	1.52 (1.16–1.97)
MDK	0.061	1.11 (0.99–1.25)	**0.008**	1.25 (1.06–1.47)
TWIST	0.537	0.96 (0.85–1.09)	**0.050**	0.87 (0.75–1.00)

Bold indicates *p*-values ≤ 0.05.

## Supplementary Materials

The following are available online at https://www.mdpi.com/2072-6694/12/1/39/s1, Table S1: Assay performance and context sequences for genes in the analysis, Table S2: Gene expression signals detected and included in analysis, Table S3: Example of calculations for cut-off and imputed values.
