# Supplementary material for: Gene Expression Alterations during Development of Castration-Resistant Prostate Cancer Are Detected in Circulating Tumor Cells
Source: Cancers (Basel). 2019 Dec 21;12(1):39. doi: 10.3390/cancers12010039 (PMC7016678; doi:10.3390/cancers12010039)
Supplement: Supplementary file 1 [file cancers-12-00039-s001.pdf]

Supplementary Materials

Gene expression alterations during development of castration-resistant prostate cancer are detected in circulating tumor cells

Andreas Josefsson, Karin Larsson, Eva Freyhult, Jan-Erik Damber and Karin Welén

Table S1. Assay performance and context sequences for genes in the analysis.

Assay name	Efficiency	r2	Slope	Y-intercept	Amplicon length	Context sequence
AGR2	93%	0.9996	-3.496	37.5	245	ACTCAGAAGCTTGGACCGCATCCTAGCCGCCGACTCACACAAGGCAGGTGGGTGAGGAAATCCAGAGTTGCCATGGAGAAAATT CCAGTGTCAGCATTCTTGCTCCTTGTGGCCCTCTCCTACACTCTGGCCAGAGATACCACAGTCAAACCTGGAGCCAAAAAGGACA CAAAGGACTCTCGACCCAAACTGCCCCAGACCCTCTCCAGAGGTTGGGGTGACCAACTCATCTGGACTCAGACATATGAAGAAG CTCTATATAAATCCAAGACAAGCAACAAACCCTTGATGATTATTCATCACTTGGATGAGTGCCCACACAGTCAA
AHR	93%	0.9998	-3.507	38.8	244	CGCCAGTCGCAAGCGGCGGAAGCCGGTGCAGAAAACAGTAAAGCCAATCCCAGCTGAAGGAATCAAGTCAAATCCTTCCAAGC GGCATAGAGACCGACTTAATACAGAGTTGGACCGTTTGGCTAGCCTGCTGCCTTTCCCACAAGATGTTATTAATAAGTTGGACAA ACTTTCAGTTCTTAGGCTCAGCGTCAGTTACCTGAGAGCCAAGAGCTTCTTTGATGTTGCATTAAAATCCTCCCCTACTGAAAGAA ACGGAGGCCAGGATAACTGTAGAGCAGCAAATTTCAGAGAAGGCCT
AKR1C3	97%	0.9995	-3.388	37.9	161	CTTCAACCGCAGGCAGCTGGAGATGATCCTCAACAAGCCAGGACTCAAGTACAAGCCTGTCTGCAACCAGGTAGAATGTCATCC GTATTTCAACCGGAGTAAATTGCTAGATTTCTGCAAGTCGAAAGATATTGTTCTGGTTGCCTATAGTGCTCTGGGATCTCAACGAG ACAAACGATGGGTGGACCCGAACTCCCCGGTGCTCTTGGAGGACC
AKT2	94%	0.9989	-3.485	38.7	136	CACGCTGCCACCATGAATGAGGTGTCTGTCATCAAAGAAGGCTGGCTCCACAAGCGTGGTGAATACATCAAGACCTGGAGGCCA CGGTACTTCCTGCTGAAGAGCGACGGCTCCTTCATTGGGTACAAGGAGAGGCCCGAGGCCCCTGATCAGACTCTACCCCCCTTAA ACAACTTCTCCGTAGCAGAATGCCAGCTGATGAAGACCGAGAGGCCGCGACCCAACACCTTT
ALDH	103%	0.9982	-3.252	35.3	133	TGTCTTTAATCCTGCAACTGAGGAGGAGCTCTGCCAGGTAGAAGAAGGAGATAAGGAGGATGTTGACAAGGCAGTGAAGGCCGC AAGACAGGCTTTTCAGATTGGATCCCCGTGGCGTACTATGGATGCTTCCGAGAGGGGGCGACTATTATACAAGTTGGCTGATTTA ATCGAAAGAGATCGTCTGCTGCTGGCGACA
ANXA2R	94%	0.9995	-3.474	39.0	114	TTGGGACTGCTTTCCAGCCCTTGCTGGCGGCTGCCCGGAGTCTACTGGCAAAACGGACTCTCTCCTGGAGTCCAGAGCACCTTGGA ACCAAGTACAGCGAAGCCCACTGAGTTCAGTTGGCCGGGGACACAGAAGCAGCAAGAGGCACCCGTAGAAGAGGTGGGGCAG GCAGAGGAACCCGACAGACTCAGGCTCCAGCAGCTTCC
AR	94%	0.9994	-3.466	36.0	250	CTGGATGGATAGCTACTCCGGACCTTACGGGGACATGCGTTTGGAGACTGCCAGGGACCATGTTTTGCCCATTGACTATTACTTTC CACCCCAGAAGACCTGCCTGATCTGTGGAGATGAAGCTTCTGGGTGTCACTATGGAGCTCTCACATGTGGAAGCTGCAAGGTCTT CTTCAAAAGAGCCGCTGAAGGGAAACAGAAGTACCTGTGCGCCAGCAGAAATGATTGCACTATTGATAAATTCCGAAGGAAAA ATTGTCCATCTTGTCGTCTTCGGAAATGTTATGAAGCAGGGATGACTCTGGG
ARV7	95%	0.9996	-3.459	37.9	186	GCGCCAGCAGAAATGATTGCACTATTGATAAATTCCGAAGGAAAAATTGTCCATCTTGTCGTCTTCGGAAATGTTATGAAGCAGG GATGACTCTGGGAGAAAAATTCCGGGTTGGCAATTGCAAGCATCTCAAAATGACCAGACCCTGAAGAAAGGCTGACTTGCCTCA TTCAAAATGAGGGCTCTAGAGGGCTCTAGTGGATAGTCTGGAGAAACCTGGCGTCTGAGGCTTAGGAGCTTAGGTTTTTGCTCCTC AACACAGACTTTGACGTTGGGGTTGGGGGCTAC
AURKA	97%	0.9997	-3.397	33.4	219	AGAAAGCCGGAGTGGAGCATCAGCTCAGAAGAGAAGTAGAAATACAGTCCCACCTTCGGCATCCTAATATTCTTAGACTGTATG GTTATTTCCATGATGCTACCAGAGTCTACCTAATTCTGGAATATGCACCACTTGGAACAGTTTATAGAGAACTTCAGAAACTTTCA

						AAGTTTGATGAGCAGAGAACTGCTACTTATATAACAGAATTGGCAAATGCCCTGTCTTACTGTCATTCGAAGAGAGTTATTCATAG<br>AGACATTAAGCCAGAGAACTTACTTCTTGGATCAGCTGGAGAGCTTAAAATTGCAGATTTTGG
BCL2	96%	0.9995	-3.412	34.3	164	CTTCAGGGACGGGGTGAACTGGGGGAGGATTGTGGCCTTCTTTGAGTTCGGTGGGGTCATGTGTGTGGAGAGCGTCAACCGGGAG<br>ATGTCGCCCCTGGTGGACAACATCGCCCTGTGGATGACTGAGTACCTGAACCGGCACCTGCACACCTGGATCCAGGATAACGGA<br>GGCTGGGATGCCTTTGTGGAACTGTACGGCCCCAGCATGCGGCCTCTGTTTGATTTCTCCTGGCTGTCT
CD44_all	105%	0.9989	-3.201	32.8	102	CGCTCCGGACACCATGGACAAGTTTTGGTGGCACGCAGCCTGGGGACTCTGCCTCGTGCCGCTGAGCCTGGCGCAGATCGATTTG<br>AATATAACCTGCCGCTTTGCAGGTGTATTCCACGTGGAGAAAAATGGTCGCTACAGCATCTCTCGGACGGAGGCCGCTGACCTCT<br>GCAAGGCTTTCAATAG
CD45/PTPRC	103%	0.9997	-3.262	37.2	98	GTAACAGAGGAGGAAATTGTTCCTCGTCTGATAAGACAACAGTGGAGAAAGGACGCATGCTGTTTCTTAGGGACACGGCTGACT<br>TCCAGATATGACCATGTATTTGTGGCTTAAACTCTTGGCATTTGGCTTTGCCTTTCTGGACACAGAAGTATTTGTGACAGGGCAAA<br>GCC
CDH1_1	97%	0.9982	-3.390	33.5	219	AAACAGGATGGCTGAAGGTGACAGAGCCTCTGGATAGAGAACGCATTGCCACATACACTCTCTTCTCTCACGCTGTGTCATCCAA<br>CGGGAATGCAGTTGAGGATCCAATGGAGATTTTGATCACGGTAACCGATCAGAATGACAACAAGCCCGAATTCACCCAGGAGGT<br>CTTTAAGGGGTCTGTCATGGAAGGTGCTCTTCCAGGAACCTCTGTGATGGAGGTCACAGCCACAGACGCGGACGATGATGTGAAC<br>ACCTACAATGCCGCCATCGCTTACACCATCCTCAGCCAAGATCCTGAGCTCCCTGACAAAAATATG
CDH11	92%	0.9995	-3.526	38.4	169	GCAGATGCCGCGGGGGCCGCTCGCAGCCGCCGCTGACTTGTGAATGGGACCGGGACTGGGGCCGGGACTGACACCGCAGCGCTT<br>GCCCTGCGCCAGGGACTGGCGGCTCGGAGGTTGCGTCCACCCTCAAGGGCCCCAGAAATCACTGTGTTTTCAGCTCAGCGGCCCT<br>GTGACATTCCTTCGTGTTGTCATTTGTTGAGTGACCAATCAGATGGGTGGAGTGTGTTAC
CDH2	93%	0.9995	-3.508	38.0	209	TGACAAAGCCCCTGGATCGCGAGCAGATAGCCCGGTTTCATTTGAGGGCACATGCAGTAGATATTAATGGAAATCAAGTGGAGA<br>ACCCCATTGACATTGTCATCAATGTTATTGACATGAATGACAACAGACCTGAGTTCTTACACCAGGTTTGGAATGGGACAGTTCCT<br>GAGGGATCAAAGCCTGGAACATATGTGATGACCGTAACAGCAATTGATGCTGACGATCCCAATGCCCTCAATGGGATGTTGAGG<br>TACAGAATC
CYP11A1	92%	0.9990	-3.521	39.6	186	GGTGGCCCTGAACCAGGAGGTGATGGCTCCAGAGGCCACCAAGAACTTTTTGCCCCTGTTGGATGCAGTGTCTCGGGACTTCGTC<br>AGTGTCCTGCACAGGCGCATCAAGAAGGCGGGCTCCGGAAATTACTCGGGGGACATCAGTGATGACCTGTTCCGCTTTGCCTTTG<br>AGTCCATCACTAACGTCATTTTTGGGGAGCGCCAGGGGATGCTGGAGGAAGTAGTGAACCCCGAGGCCCAGCGATTCATTGATGC<br>CATCTACCAGATGT
CYP17A1	97%	0.9998	-3.392	37.8	146	GACACGGCCATATGCATAACAACTTCTTCAAGCTGCAGAAAAAATATGGCCCCATCTATTCGGTTCGTATGGGCACCAAGACTAC<br>AGTGATTGTCGGCCACCACCAGCTGGCCAAGGAGGTGCTTATTAAGAAGGGCAAGGACTTCTCTGGGCGGCCTCAAATGGCAAC<br>TCTAGACATCGCGTCCAACAACCGTAAGGGTATCGCCTTCGCTGACTCTGGCGCACACTGGCAGCTGCA
CYP19A1	94%	0.9997	-3.468	37.9	201	ATCGTGGTTAAAATCCAAGGTTATTTTGATGCATGGCAAGCTCTCCTCATCAAACCAGACATCTTCTTTAAGATTTCTTGGCTATAC<br>AAAAAGTATGAGAAGTCTGTCAAGGATTTGAAAGATGCCATAGAAGTTCTGATAGCAGAAAAAAGACGCAGGATTTCCACAGA<br>AGAGAAACTGGAAGAATGTATGGACTTTGCCACTGAGTTGATTTTAGCAGAGAAACGTGGTGACCTGACAAGAGAGAATGTGAA<br>CCAGTGCATA
DDR1	94%	0.9996	-3.464	37.8	209	GCCGAAGAGGCCCCGCTCCCGGGTCGGACGCCTGGGTCTGCCGGGAAGAGCGATGAGAGGTGTCTGAAGGTGGCTATTCACTGA<br>GCGATGGGGTTGGACTTGAAGGAATGCCAAGAGATGCTGCCCCCACCCCCTTAGGCCCGAGGGATCAGGAGCTATGGGACCAGA<br>GGCCCTGTCATCTTTACTGCTGCTGCTCTTGGTGGCAAGTGGAGATGCTGACATGAAGGGACATTTTGATCCTGCCAAGTGCCGCT<br>ATGCCCTGGGCATGCAGGACCGGACCATCCCAGACAGTG
EGFR	99%	0.9992	-3.345	35.8	144	AGCTCACGCAGTTGGGCACTTTTGAAGATCATTTTCTCAGCCTCCAGAGGATGTTCAATAACTGTGAGGTGGTCCTTGGGAATTTG<br>GAAATTACCTATGTGCAGAGGAATTATGATCTTTCCTTCTTAAAGACCATCCAGGAGGTGGCTGGTTATGTCCTCATTGCCCTCAA<br>CACAGTGGAGCGAATTCCTTTGGA
EMP2	100%	0.9997	-3.320	37.5	187	TCACCTCTGCAGCCTTGCTGTTCATTGCCACCGTCGACAATGCCTGGTGGGTAGGAGATGAGTTTTTGCAGATGTCTGGAGAATA<br>TGTACCAACAACACGAATTGCACAGTCATCAATGACAGCTTTCAAGAGTACTCCACGCTGCAGGCGGTCCAGGCCACCATGATCC<br>TCTCCACCATTCTCTGCTGCATCGCCTTCTTCATCTTCGTGCTCCAGCTCTTCCGCCTGAAGCAGGGAGAGAGGTTTGTCCTAA
EPCAM	100%	0.9989	-3.313	34.7	145	TGCTGGAATTGTTGTGCTGGTTATTTCCAGAAAGAAGAGAATGGCAAAGTATGAGAAGGCTGAGATAAAGGAGATGGGTGAGAT

						GCATAGGGAACTCAATGCATAACTATATAATTTGAAGATTATAGAAGAAGGGAAATAGCAAATGGACACAAATTACAAATGTGTGTGCGTGGGACGAAGACATCTTTGAAGGTCATGAGTTTGTTAGTTTAACATCATATATTTGTAA
ESR1	98%	0.9997	-3.372	37.1	131	TGGCCAGTACCAATGACAAGGGAAGTATGGCTATGGAATCTGCCAAGGAGACTCGCTACTGTGCAGTGTGCAATGACTATGCTTCAGGCTACCATTATGGAGTCTGGTCCTGTGAGGGCTGCAAGGCCTTCTTCAAGAGAAGTATTCAAGGACATAACGACTATATGTGTCCAGCCACCAACCAGTGCACCATTGATAAAAACAGGAGGAAGAGCTGC
ESR2	93%	0.9993	-3.507	38.5	217	ACACAAAGAATATCTCTGTGTCAAGGCCATGATCCTGCTCAATTCCAGTATGTACCCTCTGGTCACAGCGACCCAGGATGCTGACAGCAGCCGGAAGCTGGCTCACTTGCTGAACGCCGTGACCGATGCTTTGGTTTGGGTGATTGCCAAGAGCGGCATCTCCTCCCAGCAGCAATCCATGCGCCTGGCTAACCTCCTGATGCTCCTGTCCCACGTCAGGCATGCGAGTAACAAGGGCATGGAACATCTGCTCAACATGAAGTGCAAAAATGTGGTCCCAGTGTATG
FOLH1	98%	0.9997	-3.380	37.6	157	TGGGGCGCTGGTGCTGGCGGGTGGCTTCTTTCTCCTCGGCTTCCTCTTCGGGTGGTTTATAAAATCCTCCAATGAAGCTACTAACATTACTCCAAAGCATAATATGAAAGCATTTTTGGATGAATTGAAAGCTGAGAACATCAAGAAGTTCTTATATAATTTTACACAGATACCACATTTAGCAGGAACAGAACAAAACTTTCAGCTTGCAAAGCAAATTCAATCCCAGTG
GAPDH	102%	0.9997	-3.280	36.8	151	GCTCTCTGCTCCTCCTGTTCGACAGTCAGCCGCATCTTCTTTTGCGTCGCCAGCCGAGCCACATCGCTCAGACACCATGGGGAAGGTGAAGGTCGGAGTCAACGGATTTGGTCGTATTGGGCGCCTGGTCACCAGGGCTGCTTTTAACTCTGGTAAAGTGGATATTGTTGCCATCAATGACCCCTTCATTGACCTCAACTACATGGTTTACATGT
GUSB	100%	0.9996	-3.317	37.1	165	CTGCCGCAGTTCTTCAACAACGTTTCTCTGCATCACCACATGCAGGTGATGGAAGAAGTGGTGCGTAGGGACAAGAACCACCCCGCGGTCGTGATGTGGTCTGTGGCCAACGAGCCTGCGTCCCACCTAGAATCTGCTGGCTACTACTTGAAGATGGTGATCGCTCACACCAAATCCTTGGACCCCTCCCGGCCTGTGACCTTTGTGAGCAACTCTAACTATGCAGCAGACAAGGGGGCTCCGTATGTGGATGTGATCT
HER2 (ERBB2)	97%	0.9990	-3.402	38.3	132	ATCTGGAAGTTTCCAGATGAGGAGGGCGCATGCCAGCCTTGCCCCATCAACTGCACCCACTCCTGTGTGGACCTGGATGACAAGGGCTGCCCCGCCGAGCAGAGAGCCAGCCCTCTGACGTCCATCATCTCTGCGGTGGTTGGCATTCTGCTGGTCGTGGTCTTGGGGGTGGTCTTTGGGATCCTCATCAAGCGACGGCAGCAGAAGATCCGGAAGTACACGA
KLK3	96%	0.9997	-3.430	37.2	217	CCTGCACCCGGAGAGCTGTGTCACCATGTGGGTCCCGGTTGTCTTCCTCACCCTGTCCGTGACGTGGATTGGTGCTGCACCCCTCATCCTGTCTCGGATTGTGGGAGGCTGGGAGTGCGAGAAGCATTCCCAACCCTGGCAGGTGCTTGTGGCCTCTCGTGGCAGGGCAGTCTGCGGCGGTGTTCTGGTGCACCCCCAGTGGGTCCTCACAGCTGCCCACTGCATCAGGAACAAAAGCGTGATCTTGCTGGGTCGGCACAGCCTGTTTCATCCTGAAGA
KRT19	98%	0.9993	-3.378	38.0	133	CCTGGCCTCCTACCTGGACAAGGTGCGCGCCCTGGAGGCGGCCAACGGCGAGCTAGAGGTGAAGATCCGCGACTGGTACCAGAAGCAGGGGCCTGGGCCCTCCCGCGACTACAGCCACTACTACACGACCATCCAGGACCTGCGGGACAAGATTCTTGGTGCCACCATTGAGAACTCCAGGATTGTCCTGCAGATCGACAATGCCCGTCTGGCTGCAGATGACTTCC
MDK	90%	0.9994	-3.601	39.0	178	AAGAAGGAGTTTGGAGCCGACTGCAAGTACAAGTTTGAGAACTGGGGTGCGTGTGATGGGGGCACAGGCACCAAAGTCCGCCAAGGCACCCTGAAGAAGGCGCGCTACAATGCTCAGTGCCAGGAGACCATCCGCGTCACCAAGCCCTGCACCCCCAAGACCAAAGCAAAGGCCAAAGCCAAGAAAGGGAAGGGAAAGGACTAGACGCCAAGCCTGGATGCCAAGGAGCCCCTGGTGTCACATG
MET	99%	0.9995	-3.343	33.2	107	TCCTTGCGCCGCTGACTTCTCCACTGGTTCCTGGGCACCGAAAGATAAACCTCTCATAATGAAGGCCCCCGCTGTGCTTGCACCTGGCATCCTCGTGCTCCTGTTTACCTTGGTGCAGAGGAGCAATGGGGAGTGTAAAGAGGCACTAGCAAAGTCCGAGATGAATGTGAATA
MYC	94%	0.9996	-3.478	37.4	299	CGCCGCCTCAGAGTGCATCGACCCCTCGGTGGTCTTCCCCTACCCTCTCAACGACAGCAGCTCGCCCAAGTCCTGCGCCTCGCAAGACTCCAGCGCCTTCTCTCCGTCCTCGGATTCTCTGCTCTCCTCGACGGAGTCCTCCCCGCAGGGCAGCCCCGAGCCCCTGGTGCTCCATGAGGAGACACCGCCCACCACCAGCAGCGACTCTGAGGAGGAACAAGAAGATGAGGAAGAAATCGATGTTGTTTCTGTGGAAAAGAGGCAGGCTCCTGGCAAAAGGTCAGAGTCTGGATCACCTTCTGCTGGAGGCCACAGCAAACCTCCTCACAGCCCACTGGTCCTCAAGAGGTGCCACGTCTCC
POU5F1	94%	0.9996	-3.484	37.6	217	ATGTGTAAGCTGCGGCCCTTGCTGCAGAAGTGGGTGGAGGAAGCTGACAACAATGAAAATCTTCAGGAGATATGCAAAGCAGAAACCCTCGTGCAGGCCCGAAAGAGAAAGCGAACCAGTATCGAGAACCGAGTGAGAGGCAACCTGGAGAATTTGTTCCTGCAGTGCCCGAAACCCACACTGCAGCAGATCAGCCACATCGCCCAGCAGCTTGGGCTCGAGAAGGATGTGGTCCGAGTGTGGTTCTGTAACCGGCGCCAGAAGGGCAAGCGATCAAGCAG

PSCA	89%	0.9996	-3.613	39.0	272	ATAAAGTCACCTGAGGCCCTCTCCACCACAGCCCACCAGTGACCACGAAGGCTGTGCTGCTTGCCCTGTTGATGGCAGGCTTGGC CCTGCAGCCAGGCACTGCCCTGCTGTGCTACTCCTGCAAAGCCCAGGTGAGCAACGAGGACTGCCTGCAGGTGGAGAACTGCAC CCAGCTGGGGGAGCAGTGCTGGACCGCGCGCATCCGCGCAGTTGGCCTCCTGACCGTCATCAGCAAAGGCTGCAGCTTGAACTG CGTGGATGACTCACAGGACTACTACGTGGGCAAGAAGAACATCACGTGCTGTGACACCGACTTGTGCAACGCCAGCGGGGCCCA TGCCCTGCAGCCGGCTGCTGCCATCCTTGCGCTGCTCC
PTCH1	92%	0.9997	-3.522	39.0	211	GGACCGGGACTATCTGCACCGGCCCAGCTACTGCGACGCCGCCTTCGCTCTGGAGCAGATTTCCAAGGGGAAGGCTACTGGCCG GAAAGCGCCGCTGTGGCTGAGAGCGAAGTTTCAGAGACTCTTATTTAAACTGGGTTGTTACATTCAAAAAAACTGCGGCAAGTTC TTGGTTGTGGGCCTCCTCATATTTGGGGCCTTCGCGGTGGGATTAAAAGCAGCGAACCTCGAGACCAACGTGGAGGAGCTGTGGG TGGAAGTTGGAGGACGAGTAAGTCGTGAATT
RUNX2	92%	0.9997	-3.535	38.3	190	AAACCAAGTAGCAAGGTTCAACGATCTGAGATTTGTGGGCCGGAGTGGACGAGGCAAGAGTTTCACCTTGACCATAACCGTCTTC ACAAATCCTCCCCAAGTAGCTACCTATCACAGAGCAATTAAAGTTACAGTAGATGGACCTCGGGAACCCAGAAGGCACAGACAG AAGCTTGATGACTCTAAACCTAGTTTGTTCTCTGACCGCCTCAGTGATTTAGGGCGCATTCCTCATCCCAGTATGAGAGTAGGTGT CCCGCCTCAGAACCCACGGCCCT
SNAI1	94%	0.9994	-3.473	38.7	170	ATTCATTGCGCCGCGGCACGGCCTAGCGAGTGGTTCTTCTGCGCTACTGCTGCGCGAATCGGCGACCCCAGTGCCTCGACCACTAT GCCGCGCTCTTTCCTCGTCAGGAAGCCCTCCGACCCCAATCGGAAGCCTAACTACAGCGAGCTGCAGGACTCTAATCCAGAGTTT ACCTTCCAGCAGCCCTACGACCAGGCCCACCTGCTGGCAGCCATCCCACCTCCG
SPINK1	94%	0.9998	-3.481	38.2	157	GGCATCTTTCTTCTCAGTGCCTTGGCCCTGTTGAGTCTATCTGGTAACACTGGAGCTGACTCCCTGGGAAGAGAGGCCAAATGTTA CAATGAACTTAATGGATGCACCAAGATATATGACCCTGTCTGTGGGACTGATGGAAATACTTATCCCAATGAATGCGTGTTATGTT TTGAAAATCGGAAACGCCAGACTTCTATCCTCATTCAAAAATCTGGGCCTTGCTGAGAACCAAGG
SRD5A1	97%	0.9993	-3.408	39.4	96	CTGCCGCTCTACCAGTACGCCAGCGAGTCCGCCCCGCGTCTCCGCAGCGCGCCCAACTGCATCCTCCTGGCCATGTTCCTCGTCCA CTACGGGCATCGGTGCTTAATTTACCCATTTCTGATGCGAGGAGGAAAGCCTATGCCACTGTTGGCGTGTACAATGGCGATTATGT TCTGTACCTGTAACGGCTATTTGC
TACSTD2	94%	0.9997	-3.468	38.8	128	GCGCGGAGAACCCCTGCAGGTGGAGCGCACGCTCATCTATTACCTGGACGAGATTCCCCCGAAGTTCTCCATGAAGCGCCTCACC GCCGGCCTCATCGCCGTCATCGTGGTGGTCGTGGTGGCCCTCGTCGCCGGCATGGCCGTCCTGGTGATCACCAACCGGAGAAAGT CGGGGAAGTACAAGAAGGTGGAGATCAAGGAACTGGGGGAGTTGAGAA
TOP2A	99%	0.9994	-3.348	36.8	176	AAAAAGGAAAGAAGAGAAATCCCTGGTCTGATTCAGAATCAGATAGGAGCAGTGACGAAAGTAATTTTGATGTCCCTCCACGAG AAACAGAGCCACGGAGAGCAGCAACAAAAACAAAATTCACAATGGATTTGGATTCAGATGAAGATTTCTCAGATTTTGATGAAA AAACTGATGATGAAGATTTTGTCCCATCAGATGCTAGTCCACCTAAGACCAAAACTTCCCCAAAACTTAGTAACAAAGAACTGA AACCAC
TP53	99%	0.9996	-3.347	36.8	103	ATCCTAGCGTCGAGCCCCCTCTGAGTCAGGAAACATTTTCAGACCTATGGAAACTACTTCCTGAAAACAACGTTCTGTCCCCCTTG CCGTCCCAAGCAATGGATGATTTGATGCTGTCCCCGGACGATATTGAACAATGGTTCACTGAAGACCCAGGTCCAGATGAAGCTC CCAGAATG
TUBB3	93%	0.9998	-3.497	37.7	164	ATCGTGCACATCCAGGCCGGCCAGTGCGGCAACCAGATCGGGGCCAAGTTCTGGGAAGTCATCAGTGATGAGCATGGCATCGAC CCCAGCGGCAACTACGTGGGCGACTCGGACTTGCAGCTGGAGCGGATCAGCGTCTACTACAACGAGGCCTCTTCTCACAAGTAC GTGCCTCGAGCCATTCTGGTGGACCTGGAACCCGGAACCATGGACAGTGTCCGCTCAGGGGCCTTTGGACATCTCTTCAG
Twist1	95%	0.9994	-3.446	38.1	145	TCCCACTAGCAGGCGGAGCCCCCCACCCCCTCAGCAGGGCCGGAGACCTAGATGTCATTGTTTCCAGAGAAGGAGAAAATGGAC AGTCTAGAGACTCTGGAGCTGGATAACTAAAAATAAAAATATATGCCAAAGATTTTCTTGGAAATTAGAAGAGCAAAATCCAAA TTCAAAGAAACAGGGCGTGGGGCGCACTTTTAAAAGAGAAAGCGAGACAGGCCCGTGGACAGTGA
UPA	101%	0.9998	-3.293	37.3	82	ATCAGCCCTTGCTGGGTGATCAGCGCCACACACTGCTTCATTGATTACCCAAAGAAGGAGGACTACATCGTCTACCTGGGTCGCT CAAGGCTTAACTCCAACACGCAAGGGGAGATGAAGTTTGAGGTGGAAAACCTCA
VEGFA	100%	0.9995	-3.330	37.0	165	CTCACCAAGGCCAGCACATAGGAGAGATGAGCTTCCTACAGCACAACAAATGTGAATGCAGACCAAAGAAAGATAGAGCAAGA CAAGAAAATCCCTGTGGGCCTTGCTCAGAGCGGAGAAAGCATTTGTTTGTACAAGATCCGCAGACGTGTAAATGTTCCTGCAAAA ACACAGACTCGCGTTGCAAGGCGAGGCAGCTTGAGTTAAACGAACGTACTTGCAGATCTCTCACCAGGAAAGACTGATACAG

Table S2. Gene expression signals detected and included in analysis.

Gene	Before ADT (*n* = 40) (A)		Signals at A included in survival analysis **	Imputed values included in survival analyses	% imputed values in survival analyses
	Detected signals	Substituted* missing signals			
AGR2	30	8	33	3	9.09
AHR	13	24	24	11	45.8
AKR1C3	23	17	31	8	25.8
AR	23	18	29	6	20.7
ARV7	9	26	19	10	52.6***
AURKA	21	19	31	10	32.2
BCL2	16	23	30	14	46.7
CDH1	22	14	26	4	15.4
CDH2	4	30	13	9	69.2***
DDR1	13	23	27	14	51.8***
EGFR	12	9	15	3	20.0
EMP2	22	16	27	5	18.5
EPCAM	36	2	37	1	2.7
FOLH1	30	7	32	2	6.25
UPA	11	25	26	15	57.7***
HER2	24	16	28	4	14.3
KLK3	33	5	33	0	0.0
KRT19	28	9	31	3	9.7
MDK	29	11	32	3	9.4
POU5F1	19	20	29	10	34.5
PSCA	17	20	25	8	32.0
SNAI1	10	19	17	7	41.2
SPINK1	20	20	28	8	28.6
SRD5A1	25	15	33	8	24.2
TACSTD2	29	9	30	1	3.3
TOP2A	26	13	29	3	10.3
TP53	34	6	37	3	8.1
TUBB3	21	15	26	5	19.2
TWIST1	21	17	26	5	19.2
VEGFA	24	16	27	3	11.1

* Missing signals in samples with too low CTC load for individual genes to be detected are not substituted. ** Only substituted values in samples with a mCq one Cq lower (ie. higher CTC content) than the mCq for the sample with the highest mCq in which the gene could be detected (mCq(h,g)). *** Samples with more than 50% imputed values were excluded from Cox regression analysis.

Supplement Table S3. Example of calculations for cut-off and imputed values.

Cq values for 5 genes and 6 samples.

	G1	G2	G3	G4	G5
S1	15.9	19.0	10.2	13.1	16.2
S2	15.2	27.4	11.9	13.8	17.2
S3	24.2		11.8	13.8	16.7
S4			12.4	16.0	18.7
S5		19.9	14.0	16.6	
S6	18.2		12.9	15.4	18.3

For each gene g, compute the maximum Cq value, Cqmax,g.

	G1	G2	G3	G4	G5
S1	15.9	19.0	10.2	13.1	16.2
S2	15.2	27.4	11.9	13.8	17.2
S3	24.2		11.8	13.8	16.7

	G1	G2	G3	G4	G5
S4			12.4	16.0	18.7
S5		19.9	14.0	16.6	
S6	18.2		12.9	15.4	18.3
Cqmax	24.2	27.4	14.0	16.6	18.7

Impute missing values for gene g with Cqmax,g +1 and compute mean Cq, mCq, for each sample.

	G1	G2	G3	G4	G5	mCq
S1	15.9	19.0	10.2	13.1	16.2	14.88
S2	15.2	27.4	11.9	13.8	17.2	17.10
S3	24.2	28.4	11.8	13.8	16.7	18.98
S4	25.2	28.4	12.4	16.0	18.7	20.14
S5	25.2	19.9	14.0	16.6	19.7	19.08
S6	18.2	28.4	12.9	15.4	18.3	18.64
Cqmax	24.2	27.4	14.0	16.6	18.7	

Compute ΔCq=Cq−mCq, but only for detectable.

	G1	G2	G3	G4	G5
S1	1.02	4.12	-4.68	-1.78	1.32
S2	-1.90	10.30	-5.20	-3.30	0.10
S3	5.22		-7.18	-5.18	-2.28
S4			-7.74	-4.14	-1.44
S5		0.82	-5.08	-2.48	
S6	-0.44		-5.74	-3.24	-0.34

For gene g, a cut off is calculated for when to consider a sample with a missing value to have sufficiently many cells to be able to impute the ΔCq: cutoff−mCqg=mCqh,g−(Cqh,g−Cqmax,g)/2, where mCqh,g=Cqmax,g is the mCq for sample h and Cqh,g is the Cq for gene g in sample h.

	G1	G2	G3	G4	G5	mCq
S1	15.9	19.0	10.2	13.1	16.2	14.88
S2	15.2	27.4	11.9	13.8	17.2	17.10
S3	24.2	28.4	11.8	13.8	16.7	18.98
S4	25.2	28.4	12.4	16.0	18.7	20.14
S5	25.2	19.9	14.0	16.6	19.7	19.08
S6	18.2	28.4	12.9	15.4	18.3	18.64
Cqmax	24.2	27.4	14.0	16.6	18.7	
h	3	5	4	4	4	
mCqh	18.98	19.08	20.14	20.14	20.14	
cutoffmCq	18.98	22.83	20.94	20.44	20.14	

Using this cut off, a missing value is only imputed and included in the analysis if its sample mCq was lower or equal to the cut off.

	**G1**	**G2**	**G3**	**G4**	**G5**
S1	1.02	4.12	-4.68	-1.78	1.32
S2	-1.90	10.30	-5.20	-3.30	0.10
S3	5.22	11.30	-7.18	-5.18	-2.28
S4		11.30	-7.74	-4.14	-1.44
S5		0.82	-5.08	-2.48	2.32
S6	-0.44	11.30	-5.74	-3.24	-0.34

For gene G1 the missing values for sample S4 and S5 are not imputed as 20.14>18.98 and 19.08>18.98, respectively.

© 2019 by the authors. Licensee MDPI, Basel, Switzerland. This article is an open access article distributed under the terms and conditions of the Creative Commons Attribution (CC BY) license (http://creativecommons.org/licenses/by/4.0/).
